# Arsenic: A Roadblock to Potential Animal Waste Management Solutions

**DOI:** 10.1289/ehp.7834

**Published:** 2005-05-12

**Authors:** Keeve E. Nachman, Jay P. Graham, Lance B. Price, Ellen K. Silbergeld

**Affiliations:** 1 Department of Health Policy and Management, and; 2 Department of Environmental Health Sciences, Johns Hopkins University, Baltimore, Maryland, USA

**Keywords:** arsenic, biomass burning, fertilizer, incineration, pelletization, poultry litter, poultry waste, waste management, waste-to-energy

## Abstract

The localization and intensification of the poultry industry over the past 50 years have incidentally created a largely ignored environmental management crisis. As a result of these changes in poultry production, concentrated animal feeding operations (CAFOs) produce far more waste than can be managed by land disposal within the regions where it is produced. As a result, alternative waste management practices are currently being implemented, including incineration and pelletization of waste. However, organic arsenicals used in poultry feed are converted to inorganic arsenicals in poultry waste, limiting the feasibility of waste management alternatives. The presence of inorganic arsenic in incinerator ash and pelletized waste sold as fertilizer creates opportunities for population exposures that did not previously exist. The removal of arsenic from animal feed is a critical step toward safe poultry waste management.

The United States produces approximately 8.5 billion broiler chickens annually (U.S. Department of Agriculture 2004), providing an unprecedented range of relatively low cost meat products for consumers worldwide. These production figures have been achieved by extraordinary changes and intensification in poultry production methods that have incidentally created a largely ignored crisis in environmental management. Every chicken produces between 1.46 and 2.67 kg of waste in its life span (Miner et al. 2000; Sharpe et al. 2004), resulting in an annual total of between 12 and 23 billion kilograms. Current federal and state regulations permit largely unrestricted land disposal of animal house wastes, which include excreta, house litter, animal carcasses, and spilled food. This practice is no longer sustainable given the dramatic changes in poultry production in the United States over the past 50 years. Because the number of farms producing livestock and poultry has dropped more than 80%, despite increasing production (Miner et al. 2000), there is now a significant concentration of animals within a given farm [or concentrated animal feeding operation (CAFO)] as well as an increased localization of these CAFOs within relatively few regions of the United States. For example, nearly 7% of U.S. broiler production takes place on the Delmarva Peninsula (Delaware, Maryland, and Virginia), with nearly 600 million chickens producing approximately 1 billion kilograms of poultry waste annually. As a result, CAFOs produce far more waste than can be managed by land disposal within the regions where it is produced. Attention has been paid to the ecologic impacts of this land disposal. When rates of land application exceed soil uptake capacity, the resulting runoff contributes to surface water eutrophication and sudden toxic algal blooms in the Chesapeake Bay and elsewhere.

However, much less attention has been given to the potential risks related to poultry waste constituents, including pathogenic bacteria, antibiotic-resistant bacteria, and residues of the drugs added to poultry feeds. Arsenic in waste results from the use of arsenicals added to poultry feed for growth promotion and prevention of parasitic infections. The U.S. Geological Survey has calculated, based on arsenic concentrations measured in poultry waste, that between 250,000 and 350,000 kg arsenic is annually applied to land in the United States (Rutherford et al. 2003). Although roxarsone, the predominant arsenical added to poultry feed, is an organoarsenical, there is strong evidence that the drug is converted into inorganic arsenic within the chicken (Arai et al. 2003) and is also rapidly transformed into inorganic arsenic in wastes and soils (Garbarino et al. 2003). Elevations in soil arsenic levels have been reported in fields where poultry wastes have been applied (Gupta and Charles 1999). This form of arsenic is readily leachable and may therefore move into groundwater (Rutherford et al. 2003).

Management of the increasing volume of poultry wastes is now being recognized as a serious challenge (Ribaudo et al. 2003), and alternatives to land amendment are being proposed, and in some cases, actively implemented. Two of these proposed alternatives, use as fuel for biomass energy plants and pelletization, are currently in commercial operation and will be expanding. Because of this, there is real urgency for a thorough examination of these solutions.

Three biomass-fueled power plants owned by Energy Power Resources (EPR) are currently in operation in the United Kingdom, and several are planned for the United States. Existing incinerators burn 680 million kilograms of poultry litter each year, and ash from the incineration process is sold as fertilizer. Fibrophos, a subsidiary of EPR, reported sales of > 63,000 metric tons of incinerator ash fertilizer between 2004 and 2005 (EPR 2005). The other new method of disposal technology is to produce fertilizer pellets directly from the waste by drying and pelletizing it. This is currently being implemented in Delaware at a relatively low rate of 55 million kilograms pellets annually (Parker 2001). A partnership has been formed between a major poultry producer and Scotts (Maysville, OH), the nation’s leading source of consumer garden products (The Scotts Company 2005), so that these pellets will be used not only in crop production but also for golf courses, landscaping, and home gardening. The use of these pellets in such settings will create a variety of opportunities for human exposures to arsenic.

Arsenic is a roadblock to potential solutions to the animal waste management crisis. Although biosolid incineration can potentially reduce or eliminate harmful pathogens in wastes (including pathogens that are resistant to antibiotics) and pelletizing processes can also in theory reduce the microbiologic risks of CAFO wastes, neither of these technologies can destroy or detoxify arsenic. Moreover, there is reason to be concerned that these new solutions to an old problem may well increase human exposures to arsenic either through air emissions from waste-to-energy plants or through contamination of soils, water, and food crops through the use of arsenic-contaminated fertilizer products. It is well known that crops grown in arsenic-contaminated soils can accumulate arsenic (Warren et al. 2003). There have been no measurements of air concentrations of arsenic at or near poultry waste incinerators. Preliminary measurements of arsenic concentrations in pelletized waste sold as fertilizer showed levels between 18 and 22 mg/kg (Chesapeake Bay Foundation, personal communication), similar to those reported in unprocessed poultry waste (Jackson and Bertsch 2001).

Arsenic is recognized as a human carcinogen by the U.S. Environmental Protection Agency (EPA), National Research Council (NRC), International Agency for Research on Cancer, National Toxicology Program, and American Conference of Industrial Hygienists, and exposures have also been associated with increased risks of heart disease, diabetes, neurologic effects, and birth defects in humans. A comprehensive reassessment of health risks of arsenic performed by the NRC in 2001 (NRC 2001) formed the basis for a recent regulatory decision by the U.S. EPA to lower the maximum contaminant level for drinking water by 5-fold (U.S. EPA 2001). As noted by Arai et al. (2003), this action must raise concerns about land disposal of arsenic-laden poultry wastes because of the likelihood of ground-water contamination.

Clearly, actions are urgently needed to deal with the increasing burden of poultry wastes from CAFOs. Existing regulations for animal waste disposal are ill-equipped to address the variety of health threats presented by poultry waste; current policies are focused on nutrient content and, as a result, do not take into account the presence of pharmaceuticals, pathogens, and heavy metals in waste. Animal waste is currently not classified as hazardous waste by the U.S. EPA. If animal waste were classified as hazardous waste, it would be prohibited from land disposal based solely on its concentrations of leachable arsenic (Rutherford et al. 2003; U.S. EPA 2004). Given the problems associated with the hazardous constituents of poultry wastes, land disposal is not a viable option. Many of these problems have been addressed in the European Union, where arsenicals were withdrawn from the poultry production process in 1998. Economic analyses have demonstrated that removal of growth-promoting antimicrobials, such as arsenic, has come at no net cost for the poultry industry [World Health Organization (WHO) 2003]. The removal of arsenic from animal feed is a critical step toward safe poultry waste management. In addition, this step will enhance food safety by reducing concentrations of arsenic in poultry products, a potentially significant source of total arsenic exposure for Americans (Lasky et al. 2004; Silbergeld 2004).

## Correction

In the manuscript originally published online, the reported sales of incinerator ash fertilizer by Fibrophos were given for 2002 and 2003; the sales have been updated here for 2004 and 2005.
